# Risk factors of perinatal depression in women: a systematic review and meta-analysis

**DOI:** 10.1186/s12888-021-03684-3

**Published:** 2022-01-27

**Authors:** Kai Yang, Jing Wu, Xiangdong Chen

**Affiliations:** Department of Anesthesiology, Union Hospital, Tongji Medical College Huazhong University of Science and Technology, Wuhan, 430022 China

**Keywords:** Perinatal depression, Risk factors, Perinatal Care, Meta-analysis

## Abstract

**Background:**

Perinatal depression in women is associated with high morbidity and mortality, and has attracted increasing attention. The investigation of risk factors of perinatal depression in women may contribute to the early identification of depressed or depression-prone women in clinical practice.

**Material and Methods:**

A computerized systematic literature search was made in Cochrane Library, PubMed, Web of Science, and EMBASE from January 2009 to October 2021. All included articles were published in English, which evaluated factors influencing perinatal depression in women. Based on the recommendations of the Cochrane Collaboration protocols, Review Manager 5.3 was used as a statistical platform.

**Results:**

Thirty-one studies with an overall sample size of 79,043 women were included in the review. Educational level (*P* = 0.0001, odds ratio [OR]: 1.40, 95% CI: [1.18,1.67]), economic status of families (*P* = 0.0001, OR: 1.69, 95%CI: [1.29,2.22]), history of mental illness (*P* < 0.00001, OR: 0.29, 95% CI: [0.18, 0.47]), domestic violence (*P* < 0.00001, OR: 0.24, 95% CI: [0.17,0.34]), perinatal smoking or drinking (*P* = 0.005, OR: 0.63; 95% CI [0.45, 0.87]; *P* = 0.008, OR: 0.43, 95% CI, [0.23 to 0.80]; respectively), and multiparity(*P* = 0.0003, OR: 0.74, 95% CI: [0.63, 0.87]) were correlated with perinatal depression in women. The stability of our pooled results was verified by sensitivity analysis and publication bias was not observed based on funnel plot results.

**Conclusion:**

Lower educational level, poor economic status of families, history of mental illness, domestic violence, perinatal smoking or drinking, and multiparity serve as risk factors of perinatal depression in women.

## Introduction

Perinatal depression refers to severe depressive episodes during pregnancy and/or within 12 months postpartum, and is one of the most common reproductive complications [1, 2]. The prevalence of perinatal depression in women is about 10–15% in developed countries, and a higher risk in less-developed countries [3, 4]. One research has suggested that nearly a fifth of women experience depression during pregnancy [5]. Perinatal depression has been shown associated with poorer pregnancy outcomes and long-standing emotional, social and cognitive difficulties in children [6]. Furthermore, perinatal depression in women is correlated with high morbidity and mortality, which imposes a massive burden on the affected individual, family members and society.

In the current study, a large number of studies have focused on the risk factors of perinatal depression in women. Several studies have suggested that many possible influencing factors related to perinatal depression in women, such as lower educational level, younger maternal age, smoking during the pregnancy, a history of depression, poor economic status of families, worse marriage status, adverse life events, antenatal depression and anxiety, and lack of social support, etc.[7–10]. However, the results of some studies remain controversial. For example, Furtado et al. reported that the maternal education level was not correlated with perinatal depression, while Martini et al. demonstrated that low education level was a significant negative factor in perinatal depression [11, 12]. Hence, in this study, we aim to present an overview of the risk factors of perinatal depression in women through a systemic review and meta-analysis.

## Material and Methods

### Eligibility criteria

This meta-analysis followed the PRISMA statement criteria [13], and the meta-analysis was not pre-registered. Inclusion and exclusion criteria for literature were determined prior to the study selection process and are described as follows:

Inclusion criteria:

1. English-written and officially published trials from January 2009 to October 2021;
2. Compare the effects of different factors on depression in perinatal women;
3. Available raw data of interested indicators;
4. Quantitative analysis of raw data by professional scales.
5. Exclusion criteria:
6. Duplicated or overlapping studies;
7. Insufficient scale of sample size (< 100);
8. Inappropriate article types such as case reports or reviews.

Using predefined criteria, row data were extracted by two researchers from tables, text contents and supplementary information within each included study, respectively.

## Literature retrieval

To guarantee the completeness of literature search, databases of Cochrane Library, PubMed, Web of Science, and EMBASE were carefully screened using the Search strategies ((Depressive Symptoms) OR (Depressive Symptom) OR (Symptom, Depressive) OR (Symptoms, Depressive) OR (Emotional Depression) OR (Depression, Emotional) OR (Depression)) AND ((Perinatal) OR (Perinatal period)) from January 2009 to October 2021. The literature search was conducted by two investigators independently. Abstracts, full-texts, and reference lists were elaborately examined to avoid unnecessary omission or ineligible inclusion during the retrieval process.

Methodological assessment.

The Newcastle–Ottawa Scale (NOS) was applied to qualitatively evaluate the included study [14]. The whole scale had a maximum score of 9 points and was composed of three items: selection, comparability and outcome. The study that scored more than 6 on our assessment using NOS methods were identified as a high-quality trial.

### Statistical analysis

All statistical processes are based on the Cochrane Collaboration protocols, that is, Review Manager 5.3 was used as a statistical software for quantitative analysis. The models of odds ratio along with 95% confidence interval were used to illustrate the effect sizes of the dichotomous variables. Heterogeneity of endpoints was measured by I^2^. The outcome definition of I^2^ values consisted low (< 25%), moderate (25%-50%), and high (> 50%) heterogeneity [15]. When I^2^ values were < 25%, a fixed-effects model was used, otherwise, a random-effects model was preferred in the remaining cases to adjust for potential differences across individual studies. To further explore the source of heterogeneity, we separately divided the primary endpoints into multiple subgroups based on different variables. Sensitivity analysis was presented by removing low-quality studies in order to observe the outcome stability. Publication bias was analyzed by evaluating the funnel plots. P values less than 0.05 were considered statistically significant.

## Results

### Study and patient characteristics

**Fig. 1:**
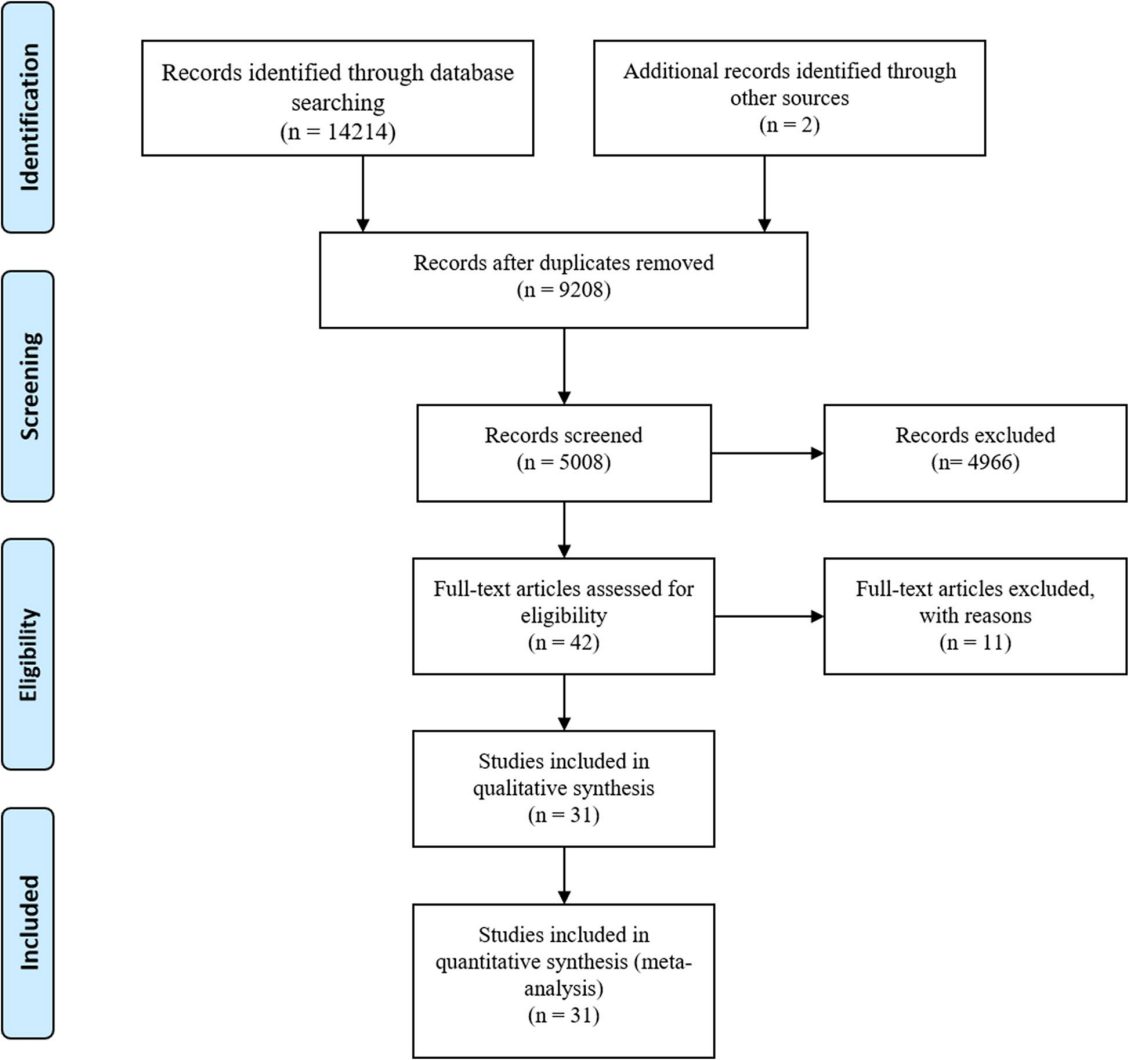
Flow diagram of study selection

Among 14,216 retrieved records, 31 studies were included in the quantitative analysis (Fig. 1). The total sample size consisted of 79,043 patients, with individual participants in each study ranging from 103 to 34,633 with a median of 564. There were 18 studies in developing countries and 13 trials in developed countries, with England accounting for the largest number of 5. Other detailed features are listed in Table 1 [16–46].

**Table 1:** Demographic information of included studies

Authors	Country	Number	Diagnostic tool	NOS scores
Alexandre 2016	Brazil	701	SRQ-20	7
Angela 2014	Canada	107	EPDS	6
Azad 2019	Bangladesh	376	EPDS	7
Bell 2015	USA	4946	EPDS	9
Chandrasekaran 2018	Canada	103	EPDS	6
Chong 2014	Singapore	709	EPDS	7
Cirik 2016	Turkey	149	HADS	6
Clarke 2014	England	9076	GHQ-12	5
Duman 2018	England	712	EPDS	7
Elias 2012	Brazil	146	EPDS	6
Gressier 2016	France	1419	ICD-10	7
Heather 2011	Canada	1403	EPDS	8
Husain 2011	England	1357	EPDS	8
Husain 2012	England	237	EPDS	7
Jane 2017	Tanzania	1013	EPDS	8
Joshi 2019	Nepal	143	EPDS	6
Katy 2009	Minneapolis	11,024	ICD-9	9
Koutra 2016	Greece	1024	EPDS	7
Li 2017	China	240	PSSS	6
Maggie 2013	England	5332	NA	5
Nyamukoho 2019	Zimbabwe	234	EPDS	7
Raghavan 2021	India	564	EPDS	7
Rurangirwa 2018	Rwanda	920	EPDS	6
Senturk 2017	Turkey	709	EPDS	7
Sheeba 2019	India	280	EPDS	6
Shi 2017	China	213	EPDS	7
Suad 2009	USA	273	NA	5
Tariq 2021	Pakistan	200	EPDS	8
Tomas 2015	Ethiopia	340	SRQ-20	8
Tong 2016	USA	34,633	NA	8
Wang 2010	Taiwan, china	460	EPDS	5

### Methodological assessment

All included studies had NOS scores of 5 to 9, and the results of evaluation comprised four 5-score studies, eight 6-score studies, eleven 7-score studies, six 8-score studies, and two 9-score studies. Of these, the studies of 5 points included wang 2010, Clarke 2014, Maggie 2013 m and Suad 2009, the important reasons for these studies of low scores were unclear evaluation methods and fewer included studies entries (Table 1).

### Social and economic factors

For this part, maternal age, pregnancy planning, marriage status, maternal education level, family economic status, and maternal employment status were included in the further meta-analysis. The results indicate that maternal education level and family economic status were the risk factors of perinatal depression, while maternal age, pregnancy planning, marriage status, and maternal employment status were not significantly associated with perinatal depression (Table 2).

**Table 2:** Meta-analysis of factors not reaching statistical significance in perinatal depression

Maternal characteristics	P value	OR	95% CI	I2
Maternal age	0.30	0.90	[0.73, 1.10]	84%
Pregnancy planning	0.09	0.73	[0.51, 1.05]	86%
Marriage status	0.60	1.09	[0.79, 1.49]	88%
Maternal employment status	0.79	1.04	[0.78, 1.39]	70%
Caesarean section	0.06	0.81	[0.64, 1.01]	63%
Premature birth	0.95	0.99	[0.73, 1.34]	47%
Fetal gender	0.65	0.96	[0.79, 1.15]	22%
Serious perinatal health problems	0.67	0.92	[0.64, 1.34]	90%
Hypertension or diabetes	0.52	0.82	[0.44, 1.52]	92%

**Fig. 2:**
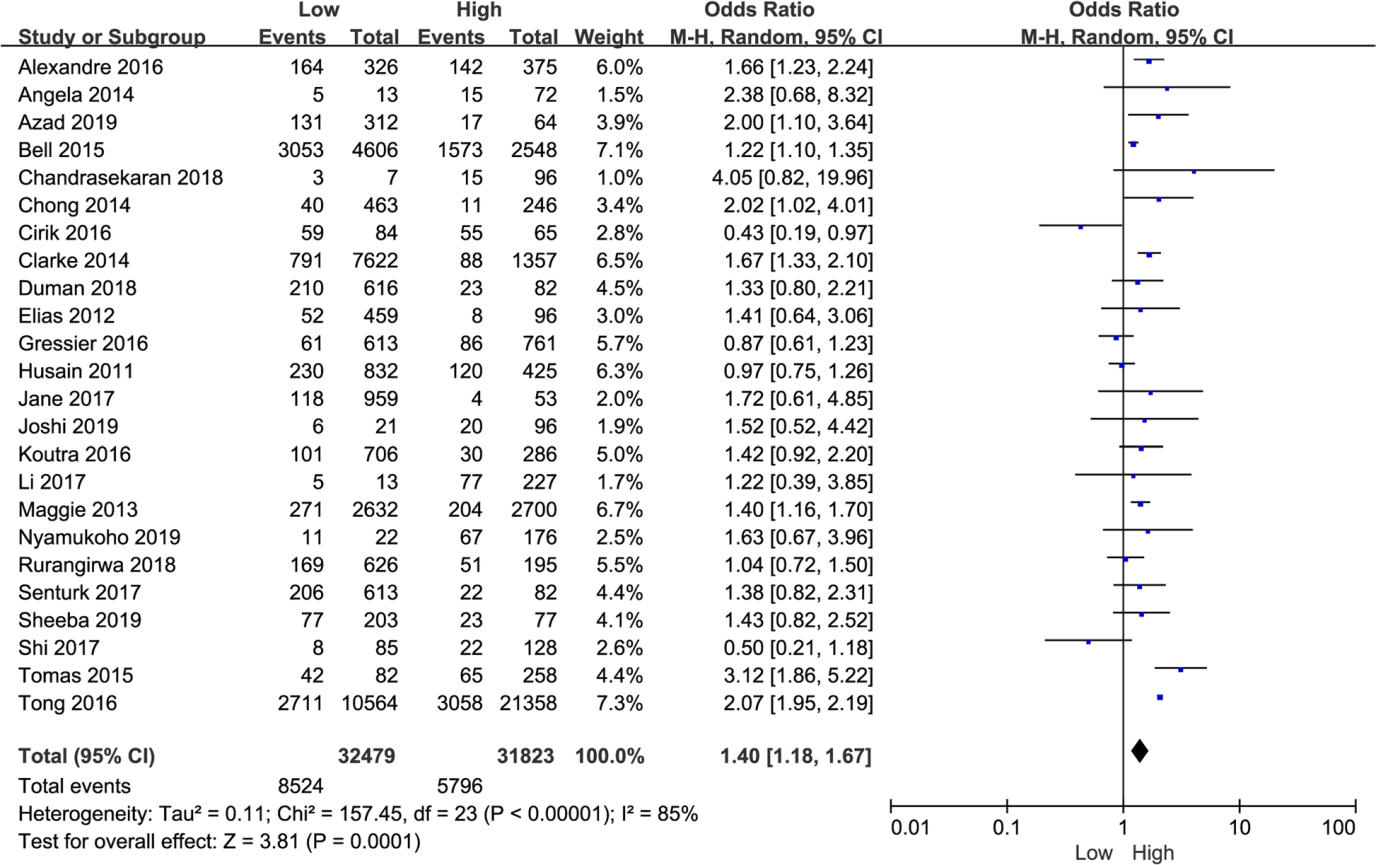
The correlation between maternal education levels and perinatal depression in women

Specifically, twenty-four studies provided row data on perinatal depression with different maternal education levels. Compared with higher education levels, perinatal women with lower education levels were significantly more susceptible to depression, along with high heterogeneity of source uncertainty (*P* = 0.0001, OR: 1.40, 95% CI: [1.18,1.67], I^2^ = 85%) (Fig. 2). Subgroup analysis by countries with different economic levels showed that perinatal women from developing nations (*P* = 0.008, OR: 1.38, 95% CI: [1.09,1.76], I^2^ = 54%) or developed country (*P* = 0.006, OR: 1.43, 95% CI: [1.11,1.84], I^2^ = 93%), lower education level were associated with increased depression (Table 3).

**Table 3:** Subgroup analysis of factors groups associated with perinatal depression in women according to country income

**Maternal characteristics**		***P* value**	**OR**	**95% CI**	**I**^**2**^
Maternal education level	developing nations	0.008	1.38	[1.09,1.76]	54%
	developed country	0.006	1.43	[1.11, 1.84]	93%
Family economic status	developing nations	0.002	1.82	[1.24, 2.66]	80%
	developed country	0.0008	1.30	[1.12, 1.51]	0%
History of mental illness	developing nations	< 0.00001	0.24	[0.16, 0.37]	69%
	developed country	0.02	0.36	[0.16, 0.82]	94%
Domestic violence	developing nations	< 0.00001	0.22	[0.14,0.32]	77%
	developed country	< 0.00001	0.44	[0.32, 0.60]	/
Parity	developing nations	0.02	0.78	[0.63,0.96]	54%
	developed country	0.005	0.67	[0.50, 0.89]	87%

**Fig. 3:**
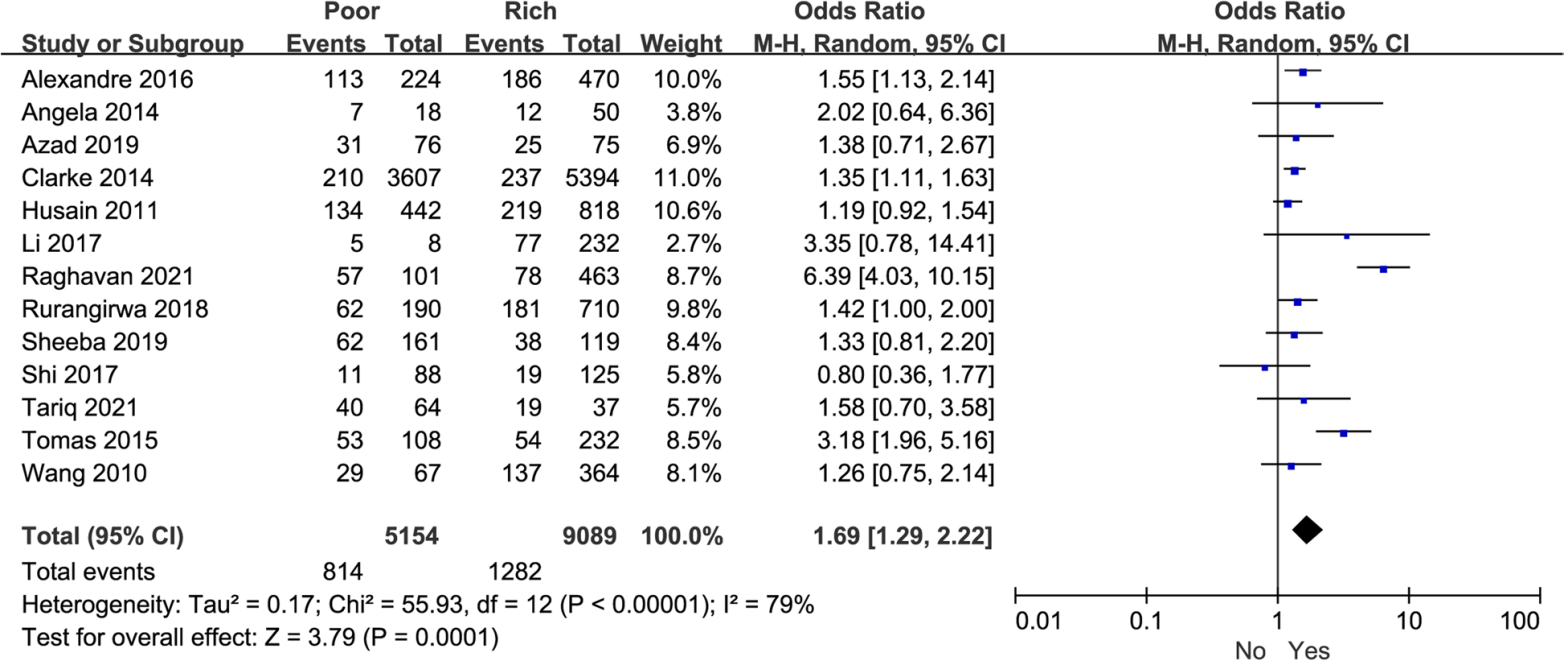
The correlation between family economic status and perinatal depression in women

In addition, raw data of perinatal depression in various family economic status was obtained from 13 studies. Our pooled results indicated that poorer family economic status was significantly correlated with depression in perinatal women (*P* = 0.0001, OR: 1.69, 95%CI: [1.29,2.22], I^2^ = 79%) (Fig. 3). As for the source regions of perinatal women with depression, poorer family economic status was significantly related to increased depression from developing nations (*P* = 0.002, OR: 1.82, 95% CI: [1.24, 2.66], I^2^ = 80%) and developed country (*P* = 0.0008, OR: 1.30, 95% CI: [1.12, 1.51], I^2^ = 0%). Regarding subgroups of different country incomes, our pooled results showed that poorer family economies were strongly associated with increased depression in perinatal women (Table 3).

### Family environment and past medical history

**Fig. 4:**
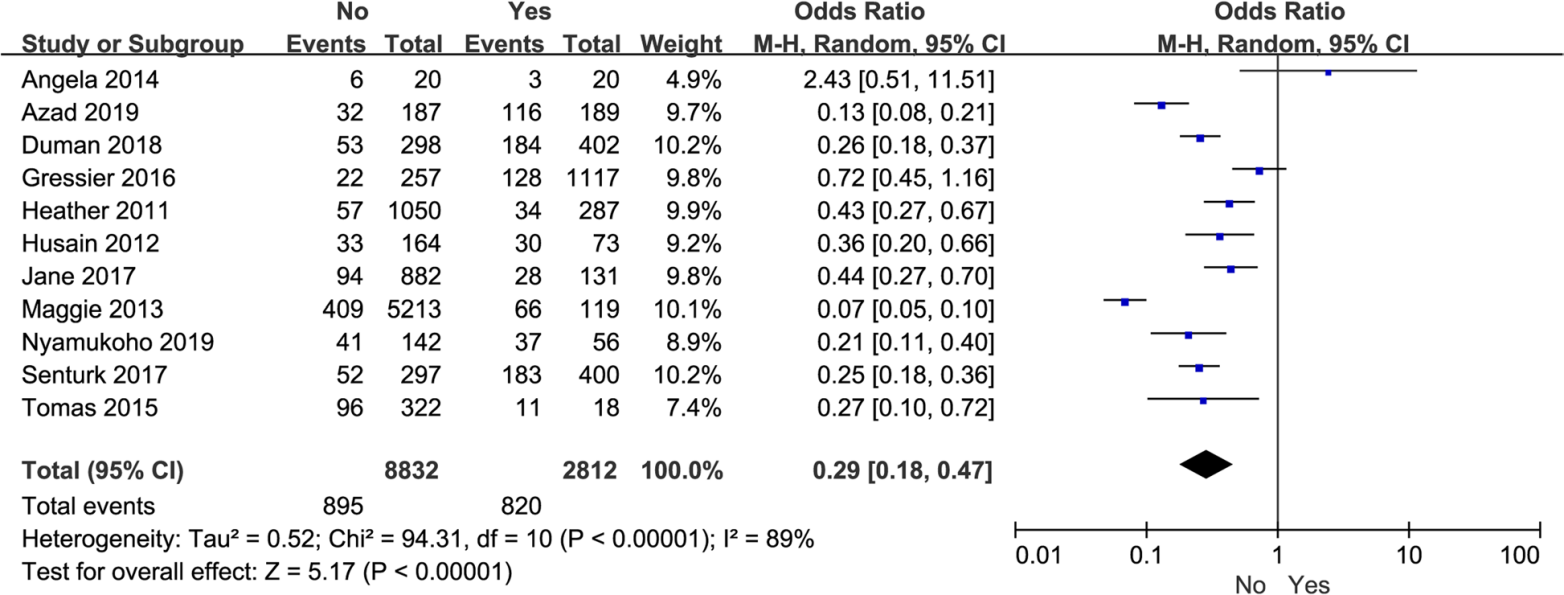
The correlation between the history of mental illness and perinatal depression in women

we next counted and evaluated the impact of the past mental health history of perinatal women and domestic violence on depression. The original data from 11 studies on the correlations between psychiatric history and perinatal depression were extracted. The pooled results showed that there was a significant correlation between the history of previous mental illness and depression in perinatal women (*P* < 0.00001, OR: 0.29, 95% CI: [0.18, 0.47]) (Fig. 4). High heterogeneity of unknown sources was observed (I^2^ = 89%). Depending on the countries with different economic levels, the included studies were divided into two subgroups. The history of previous mental illness was obviously associated with depression in perinatal women, regardless of whether perinatal women was from developing countries (*P* < 0.00001, OR: 0.24, 95% CI: [0.16, 0.37], I^2^ = 69%) or developed country (*P* = 0.02, OR: 0.36, 95% CI: [0.16, 0.82], I^2^ = 94%) (Table 3).

**Fig. 5:**
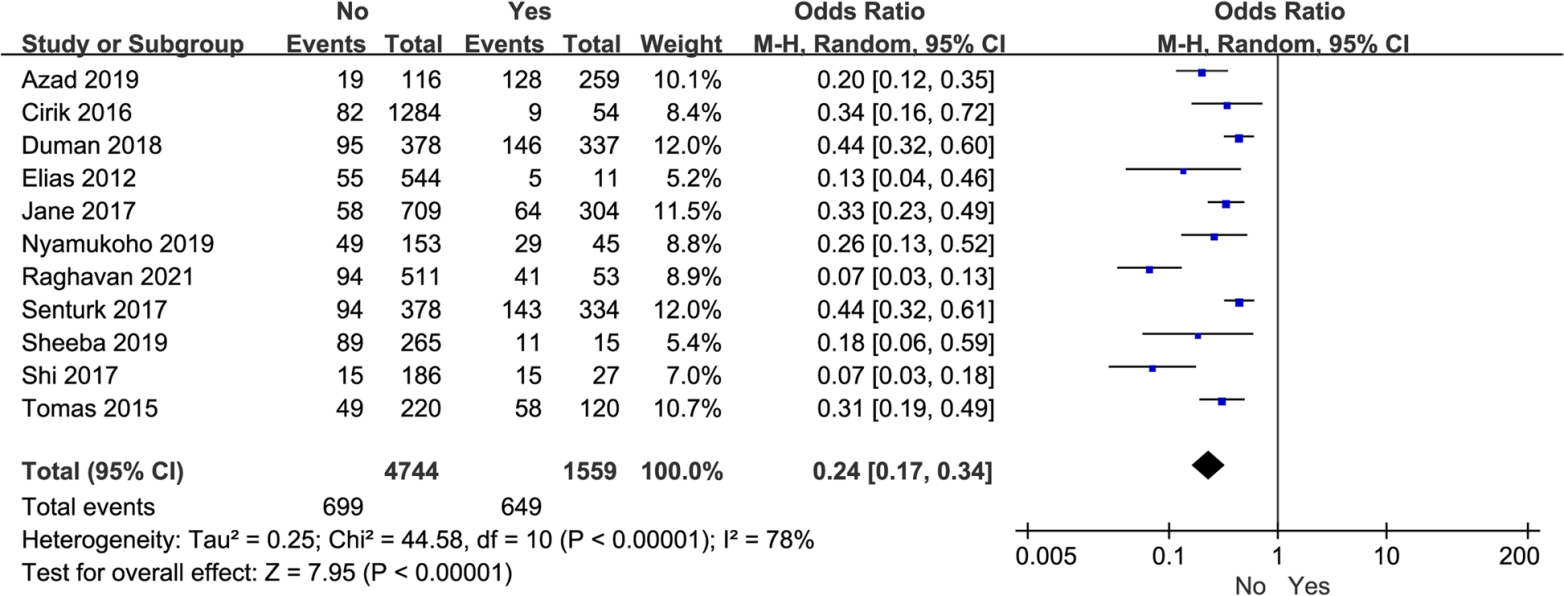
The correlation between domestic violence and perinatal depression in women

In addition, the meta-analysis of 11 studies demonstrated that domestic violence had a negative effect on depression in perinatal women (*P* < 0.00001, OR: 0.24, 95% CI: [0.17,0.34], I^2^ = 78%) (Fig. 5). Similarly, we performed a following subgroup analysis to elucidate the possible confounding factors. Based on subgroups analysis of different source regions, domestic violence in developed country (*P* < 0.00001, OR: 0.44, 95% CI: [0.32,0.60]) or developing nations (*P* < 0.00001, OR: 0.22, 95% CI: [0.14,0.32], I^2^ = 77%) contributed to a significantly depression in perinatal women (Table 3).

Maternal lifestyle.

In this part, we mainly focused on the impacts of maternal lifestyles including perinatal smoking and drinking on perinatal depression. Concerning smoking and drinking in perinatal women, only six and five studies offered original data, respectively. As a results, perinatal women with smoking (*P* = 0.005, OR: 0.63, 95% CI: [0.45,0.87]) or drinking (*P* = 0.008, OR: 0.43, 95% CI: [0.23,0.80]) were more prone to depression (Fig. 5). Moderate and high heterogeneity was observed respectively in both analyses (smoking: I^2^ = 26% and drinking: I^2^ = 66%). The subgroup analysis had not been further implemented due to fewer studies included.

### Reproductive health factors

**Fig. 6:**
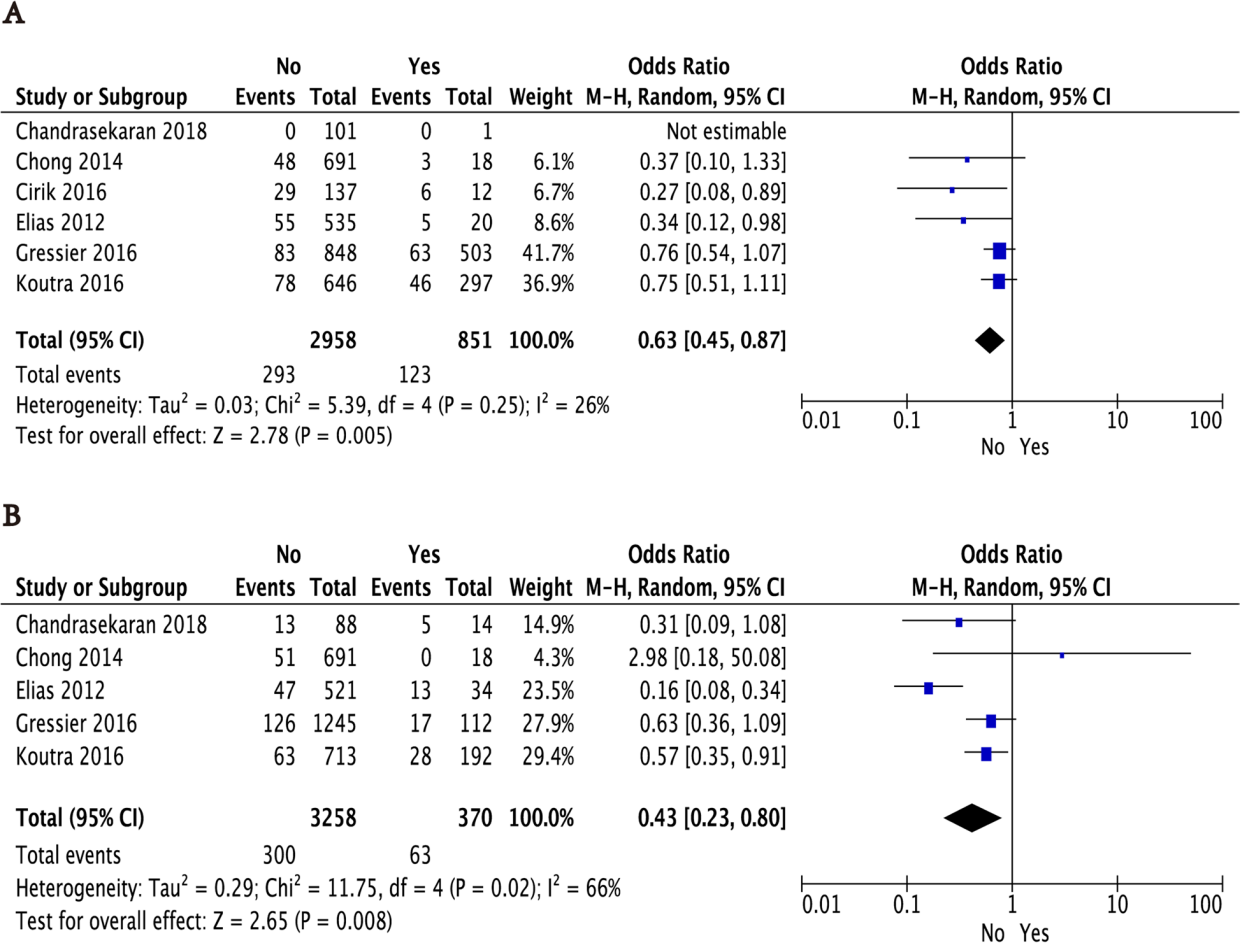
The correlation between perinatal smoking and drinking and perinatal depression in women

The original data about parity, cesarean section, premature birth, fetal gender, serious perinatal health problems (excluding hypertension or diabetes), and hypertension or diabetes were included in our meta-analysis. As shown in Table 2 and Fig. 6, the results indicated that parity was the influencing factor of perinatal depression, while cesarean section, premature birth, fetal gender, serious perinatal health problems (excluding hypertension or diabetes), and hypertension or diabetes were not significantly associated with perinatal depression.

**Fig. 7:**
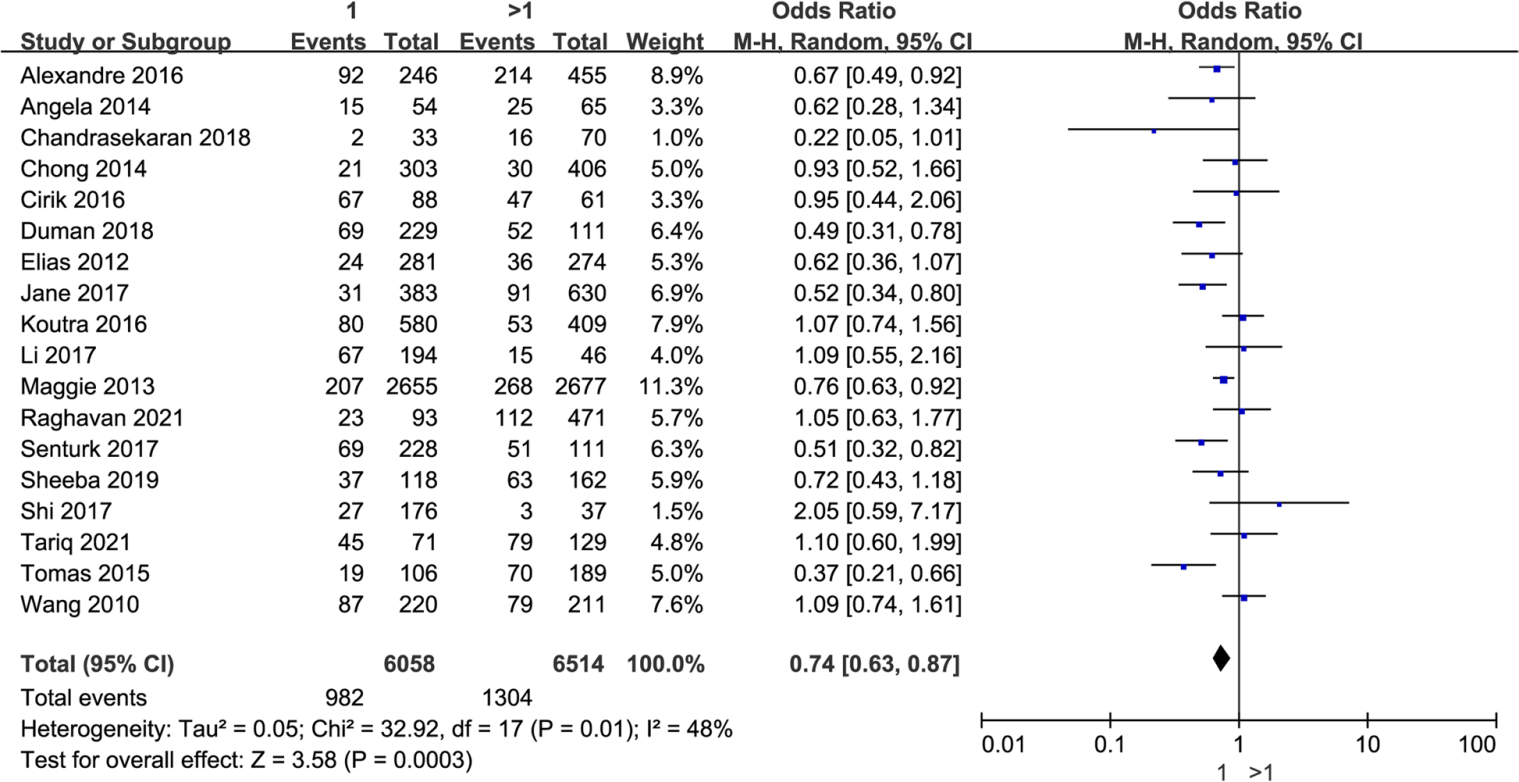
The correlation between multiparity and perinatal depression in women

Eighteen trials provided original data on depression in perinatal women in terms of parity. It demonstrated that perinatal women with multiparity were highly associated with perinatal depression (*P* = 0.0003, OR: 0.74, 95% CI: [0.63, 0.87], I2 = 48%) (Fig. 7). In the subgroup analysis by national economic level, a higher proportion of depression was observed among perinatal women with multiparity both in developing (*P* = 0.02, OR: 0.78, 95% CI: [0.63,0.96], I^2^ = 54%) and developed nations (*P* = 0.005, OR: 0.67, 95% CI: [0.50, 0.89], I^2^ = 87%) (Table 3).

## Sensitivity analysis

As shown in Table 4, the elimination of studies with 5 points on the NOS assessment was unable to affect the negative outcome of the maternal low-education levels (*P* = 0.001, OR: 1.38, 95% CI: [1.13, 1.69], I^2^ = 86%), poor family economic status (*P* = 0.001, OR: 1.80, 95% CI: [1.27, 2.56], I^2^ = 81%), history of mental illness (*P* < 0.00001, OR: 0.33, 95% CI: [0.23, 0.47], I^2^ = 77%) and multiparity (P = 0.0006, OR: 0.72, 95% CI: [0.59, 0.87], I^2^ = 48%) on depression in perinatal women. Besides, the original data of the domestic violence, perinatal smoking and drinking did not include anyone of study with NOS score of 5, so we did not further conduct sensitivity analysis in other ways.

**Table 4:** Sensitivity analysis results of factors related to perinatal depression in women after removal of poor quality studies

Maternal characteristics	*P* value	OR	95% CI	I2
Education level	0.001	1.38	[1.13, 1.69]	86%
Family economic status	0.001	1.80	[1.27, 2.56]	81%
History of mental illness	< 0.00001	0.33	[0.23, 0.47]	77%
Parity	0.0006	0.72	[0.59, 0.87]	48%

### Publication bias

**Fig. 8:**
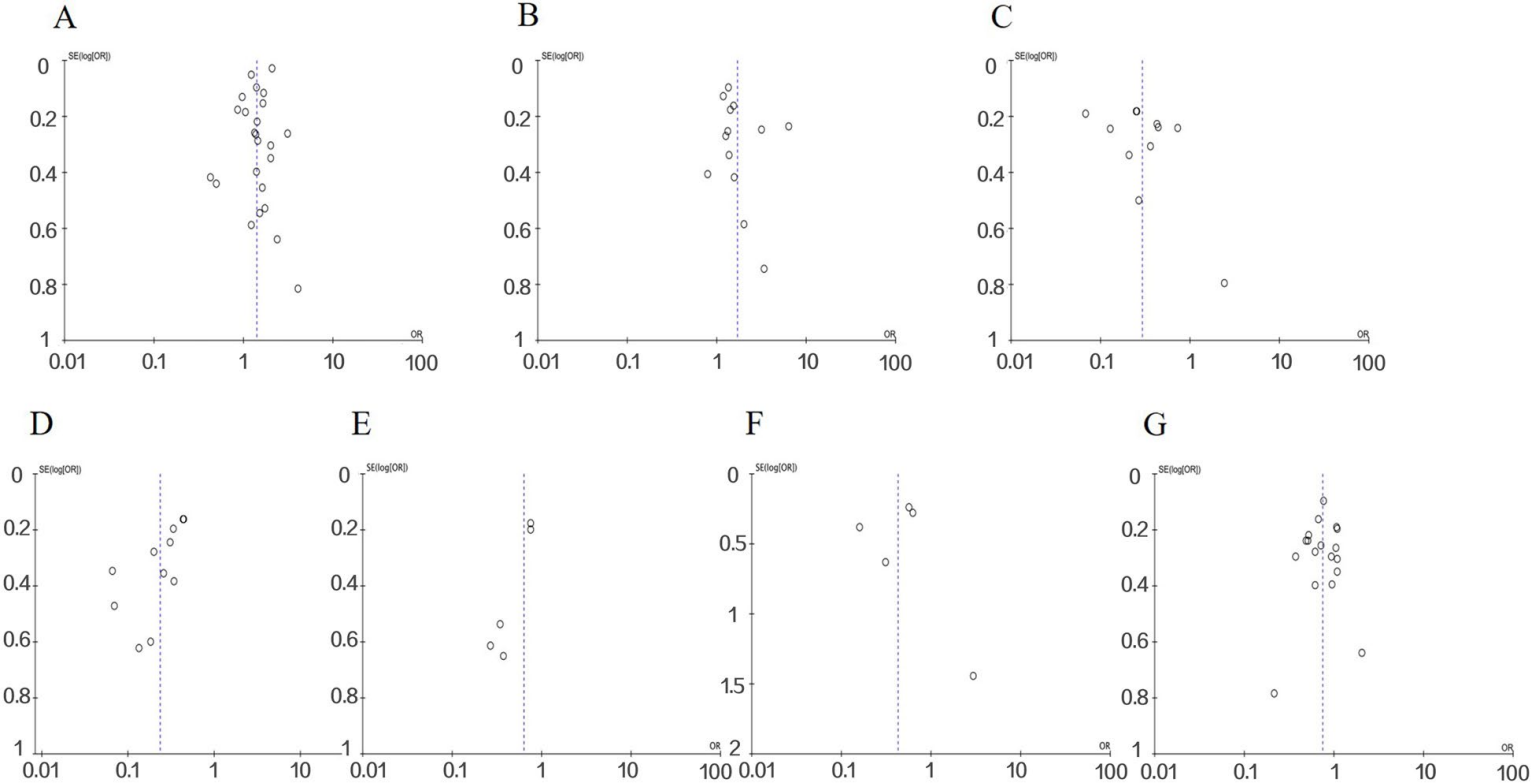
The funnel plots of this meta-analysis, including (**A**) maternal education levels, (**B**) economic status of families, (**C**) the history of mental illness, (**D**) domestic violence, (**E**) perinatal smoking, (**F**) perinatal drinking, and (**G**) parity

No obvious publication bias was observed in our meta-analysis results following funnel plots validation (Fig. 8).

## Discussion

This meta-analysis examined the risk factors of perinatal depression. We found evidence supporting lower educational level, poor economic status of families, history of mental illness, domestic violence, perinatal smoking or drinking, and multiparity were associated with depression in perinatal women, regardless of the subgroup confounding variables.

Perinatal depression is no different from general depression and presents with a state of low mood, inactivity, fatigue, sleep disturbances, disconcertment, disorientation or suicidal thinking [16]. In addition, it was associated with poorer pregnancy outcomes and long-standing emotional, social and cognitive difficulties in children [47]. Prevention and early intervention in perinatal mental health have been identified as potentially important strategies, American College of Obstetricians and Gynecologists guidelines have recommended screening for perinatal depression at least once during the perinatal period [48, 49]. And The US Preventive Services Task Force suggested that counseling interventions, such as cognitive-behavioral therapy and interpersonal therapy, are effective in preventing perinatal depression [50]. However, lacking effective methods to identify women who are prone to perinatal depression remains a major obstacle.

Previous studies focused on a single influence (disease or region) on perinatal depression [51, 52]. In our current meta-analysis, some factors have been revealed to be inextricably linked to perinatal depression in women, which include lower educational level, poor economic status of families, history of mental illness, domestic violence and multiparity. Similar to our results, domestic violence is significantly associated with perinatal depression [53, 54]. And we novelty demonstrated that perinatal smoking or drinking increased the incidence of perinatal depression. It is acknowledged that people with mental health problems are more likely to smoke/drink which may, in turn, worsen mental health conditions [55]. Importantly, tobacco cessation may contribute to improved depressive symptoms of perinatal depression women.

Although Biaggi et al. reported that pregnancy complications were a risk factor for perinatal depression, further data collection was needed to clarify whether different complications had different effects on depression to further explain these controversies [56]. Based on our results, the association between reproductive health factors and perinatal depression was not observed. Medical literature also pointed out that the conditions of the delivery room usually increase the risk of maternal depression [57]. Improving the conditions of the delivery room is also an important measure to reduce the tendency of perinatal depression, especially for some developing countries. Of course, the above results are only reported in a few studies, and they are not analyzed as our combined results. In addition, many studies have compared the effects of cesarean section and vaginal delivery on perinatal depression, but there are few studies on the relationship between auxiliary intervention during vaginal delivery and perinatal depression. We look forward to more research results to confirm the link between auxiliary interventions during vaginal delivery, such as fetal monitoring, lateral perineal incision, perineal tear, catheterization and enema, etc. and perinatal depression. This will have obvious guiding significance for whether to carry out relevant auxiliary interventions during vaginal delivery.

Apart from the significant results, our quantitative meta-analysis has some limitations. First, despite the subgroup analysis, the heterogeneity of our study was not completely eliminated, which may lead to bias in the results to a certain extent. We suspected that heterogeneity mainly came from the following aspects: 1) The evaluation criteria of each study were not uniform. 2) Some factors cannot be integrated into our analysis, such as ethnicity. Secondly, although the total sample sizes exceeded 70,000, the influencing factors of some included studies were insufficient, leading to some potential influencing factors that cannot produce meaningful results. Taken together, despite the above shortcomings, our meta-analysis demonstrates several factors associated with depression in perinatal women. This is consistent with other studies in the field, highlighting the value of screening and monitoring these indicators of maternal. We recommend that our pooled results be used to prevent and clinically guide women with perinatal depression.

## Conclusion

Perinatal women in the following cases: lower educational level, poor economic status of families, history of mental illness, domestic violence, perinatal smoking or drinking, and multiparity have a higher incidence of perinatal depression than without these conditions. Therefore, it is recommended that early screening and counseling interventions for those women to prevent perinatal depression.

## Data Availability

The data used to support the findings of this study are included within the article.
